# Bile Duct Calculi – The New Challenges

**DOI:** 10.1155/1998/16548

**Published:** 1998

**Authors:** Gregory V. Stiegmann

**Affiliations:** Department of Surgery University of Colorado School of Medicine Denver Colorado 80220 USA

## Abstract

*Background*: Morbidity and mortality after surgical treatment of bileduct stones increase with age and associated diseases. A proposed alternative therapy is endoscopic sphincterotomy (ES) with the gallbladder left in situ, and we elected to compare this option with standard open surgery in high-risk patients.

*Methods*: 98 patients (mean age 80 years) with symptoms likely to be due to bileduct stones or a recent episode of biliary parcreatitis were randomised to be treated either by open cholecystectomy with operative cholangiography and (if necessary) bileduct exploration (*n*=48) or by endoscopic sphincterotomy alone (*n*=50).

*Findings*: The procedure was accomplished successfully in 94% of the surgery group and 88% of the ES group, and there were no significant differences in immediate morbidity (23% *vs* 16%) or mortality (4% *vs* 6%). During mean follow-up of 17 months biliary symptoms recurred in three surgical patients, none of whom underwent repeat surgery, and in 10 ES patients, seven of whom had biliary surgery. By multivariate regression analysis endoscopic sphincterotomy was an independent predictor of recurrent biliary symptoms (odds ratio 6.9; 95% C11.46 to 32.54).

*Interpretation*: In elderly or high-risk patients, surgery is preferably to endoscopic sphincterotomy with the gallbladder left *in situ* as a definitive treatment for bileduct stones or non-severe biliary pancreatitis.


### Bile Duct Calculi – The New Challenges

### ABSTRACT

*Targarona, E. M., Perez Ayuso, R. M., Bordas, J. M., Ros, E., Pros, I., Martinez, J., Teres, J. and Trias, M. (1996) Randomised trial of endoscopic sphincterotomy with gallbladder left in situ versus open surgery for common bileduct calculi in high-risk patients. The Lancet;* **347**, *926–929.*

***Background:*** **Morbidity and mortality after surgical treatment of bileduct stones increase with age and associated diseases. A proposed alternative therapy is endoscopic sphincterotomy (ES) with the gallbladder left *in situ*, and we elected to compare this option with standard open surgery in high-risk patients.**
***Methods:*** **98 patients (mean age 80 years) with symptoms likely to be due to bileduct stones or a recent episode of biliary parcreatitis were randomised to be treated either by open cholecystectomy with operative cholangiography and (if necessary) bileduct exploration (*n* = 48) or by endoscopic sphincterotomy alone (*n* = 50).**
***Findings:*** **The procedure was accomplished successfully in 94% of the surgery group and 88% of the ES group, and there were no significant differences in immediate morbidity (23% *vs* 16%) or mortality (4% *vs* 6%). During mean follow-up of 17 months biliary symptoms recurred in three surgical patients, none of whom underwent repeat surgery, and in 10 ES patients, seven of whom had biliary surgery. By multivariate regression analysis endoscopic sphincterotomy was an independent predictor of recurrent biliary symptoms (odds ratio 6.9; 95% Cl 1.46 to 32.54).**
***Interpretation:*** **In elderly or high-risk patients, surgery is preferably to endoscopic sphincterotomy with the gallbladder left *in situ* as a definitive treatment for bileduct stones or non-severe biliary pancreatitis.**

*Keywords:* Gallstones, bile duct, cholecystectomy, endoscopic retrograde cholangiography

### PAPER DISCUSSION

Targarona and colleagues from Barcelona have conducted the first (and probably the last) prospective randomized trial comparing open cholecystectomy/common bile duct exploration with endoscopic sphincterotomy leaving the gallbladder *in situ*, for high-risk patients presenting with complications of bile duct calculi [1]. These two treatments produced no difference in short-term morbidity or mortality. Patients treated with endoscopic sphincterotomy had an overall 21 per cent incidence of recurrent biliary symptoms as compared with a six percent incidence for patients treated with surgery. Fifteen percent of the endoscopic sphincterotomy group enventually needed cholecystectomy during the 18 month follow up period. There was no long-term difference in survival between the groups. Thirteen deaths during the follow-up period confirmed the high risk nature of these patients. Only one of these deaths resulted from biliary causes – recurrent cholangitis in a 107 year old treated with endoscopic sphincterotomy. Little mention was made of the costs associated with the two treatments. Patients treated with endoscopic sphincterotomy spent less than half as much time in hospital following the initial treatment compared with those in the surgical cohort. One fifth of the endoscopically treated patients, however, required later re admission for biliary symptoms and/or cholecystectomy.

What can we learn from this well done trial which was conceived just as the sun was setting on conventional biliary surgery? The advantages of laparoscopic cholecystectomy have made open common duct exploration a less relevant topic now than when this study began. Today, laparotomy for common bile duct exploration is usually reserved for patients in whom laparoscopic cholecystectomy is not feasible; for those who are not candidates for or fail endoscopic stone removal; or for patients with multiple duct calculi in whom a durable biliary drainage procedure is indicated. In spite of the fact that morbidity and the chance of dying from endoscopic treatment are no less than from conventional cholecystectomy and duct exploration, the former treatment has instinctive appeal, particularly for elderly and high-risk patients. Two questions remain unanswered: 1) should patients who have endoscopic clearance of duct stones undergo subsequent elective laparoscopic cholecystectomy, 2) is the morbidity and mortality of laparoscopic bile duct exploration less than that for either endoscopic stone removal or open cholecystectomy and choledocholithotomy?

Should elective laparoscopic removal of the gallbladder be done following endoscopic clearance of duct calculi in patients without overt symptoms of cholecystitis? Neither cystic duct patency nor other indicators are reliable in predicting which patients will eventually develop symptoms related to the gallbladder. The current study reafirms that patients who present with gallstone pancreatitis and have successful endoscopic sphincterotomy have a low risk for recurrence of pancreatitis. They are, however, at significant (29 per cent in the this study) risk for developing other complications which are usually related to the gallbladder. The majority of patients with an intact gallbladder who undergo endoscopic duct clearance for symptoms related to duct calculi may not develop subsequent biliary symptoms. Therefore, the prudent approach is conservative, especially in high-risk individuals. When and if symptoms related to the gallbladder develop, cholecystectomy should not be delayed.

The efficacy and safety of laparoscopic transcystic duct exploration and stone removal, in fit patients, is established [2]. There are no studies which address the high risk patient in whom complications of common duct calculi are the presenting clinical feature. Trans-cystic duct laparoscopic removal of duct calculi has limitations, particularly when stones are multiple or large. Laparoscopic incision of the common bile duct for direct exploration and removal of stones attempts to replicate conventional open duct exploration [3]. This approach allows more effective treatment of large or multiple stones than the trans-cystic method but requires greater technical dexterity. Although the number of individuals and institutions performing laparoscopic duct exploration of either type is still small, many surgeons are seeking training in laparoscopic management of common duct calculi. These options will become widely available within the next few years.

The current study has reconfirmed that, in high risk patients, endoscopic stone removal is associated with risks similar to conventional cholecystectomy and duct exploration. Laparoscopic choledocholithotomy may be associated with less morbidity and mortality than either. Removing the diseased gallbladder simultaneously with the bile duct calculi will eliminate most instances of recurrent biliary symptoms. Laparoscopic duct exploration may prove as advantageous in terms of shortened hospitalization and rapid return to normal activity as laparoscopic cholecystectomy. Targarona *et al.* correctly conclude that "future trials should compare preoperative flexible endoscopic therapy followed by laparoscopic cholecystectomy with simultaneous laparoscopic cholecystectomy and bile duct exploration." This may be the dawn of a new age of comprehensive laparoscopic biliary tract surgery. It is time to initiate trials designed to assess these methods both in terms of traditional mortality and morbidity outcomes as well as from the quality of life and cost-benefit perspectives.

### *References*

[1] Tagarona, E. M., Perez Ayuso, R. M., Bordas, J. M., Pros, I., Martinez, J., Teres, J. and Trias, M. (1995). Randomised trial of endoscopic sphincterotomy with gallbladder left *in situ* versus open surgery for common bile duct calculi in high-risk patients. *The Lancet*, **347**, 926 –9.

[2] Strasberg, S. M. and Soper, N. J. (1995). Management of choledocholithiasis in the laparoscopic era. *Gastroenterol.*, **109**, 320 –21.

[3] Cuschieri, A., in Berci, G. and Cushieri, A. (1997). *Bile Ducts and Bile Duct Stones.* W.B. Saunders, Philadelphia., 109 –16.

Gregory V. Stiegmann, MD
Department of Surgery
University of Colorado School of Medicine
Denver, Colorado 80220

